# The effect of distress on the balance between goal-directed and habit networks in obsessive-compulsive disorder

**DOI:** 10.1038/s41398-020-0744-7

**Published:** 2020-02-24

**Authors:** Anouk van der Straten, Wieke van Leeuwen, Damiaan Denys, Hein van Marle, Guido van Wingen

**Affiliations:** 1 Amsterdam UMC, Department of Psychiatry, Amsterdam Neuroscience, University of Amsterdam, Amsterdam, Netherlands; 2 Amsterdam Brain and Cognition, University of Amsterdam, Amsterdam, Netherlands; 3 Spinoza Centre for Neuroimaging, Royal Netherlands Academy for Arts and Sciences, Amsterdam, Netherlands; 4 Department of Psychiatry, Amsterdam Neuroscience, Amsterdam UMC, Vrije Universiteit Amsterdam, Amsterdam, Netherlands

**Keywords:** Psychiatric disorders, Neuroscience

## Abstract

The classical cognitive-behavioral theory of obsessive-compulsive disorder (OCD) holds that compulsions are performed to reduce distress that is evoked by obsessions, whereas a recent neuroscience-inspired theory suggests that compulsivity results from a disbalance between goal-directed and habit-related neural networks. To bridge these theories, we investigated whether the balance between goal-directed and habit networks in patients with OCD was affected during psychological distress. Twenty-three OCD patients and twenty-three healthy controls participated in a controlled stress induction paradigm using the socially evaluated cold-pressor test in a crossover design. Stress responses were evaluated through cortisol levels, blood pressure, and anxiety ratings. Functional connectivity of the caudate nucleus and posterior putamen was assessed using seed region analysis of resting-state functional magnetic resonance imaging data, which are hubs of the goal-directed and habit network, respectively. Stress induction increased blood pressure and psychological stress measures across groups and resulted in blunted cortisol responses in patients. Furthermore, patients showed a blunted reduction in connectivity between the caudate nucleus and precuneus during psychological distress, which was positively correlated with compulsivity but not obsession severity. The posterior putamen showed no significant group differences in distress-induced connectivity. These results suggest that compulsivity in OCD is associated with altered connectivity between the goal-directed and default mode networks during psychological distress.

## Introduction

Obsessive–compulsive disorder (OCD) is a debilitating psychiatric disorder with an estimated lifetime prevalence of 1–3%^1^. Patients with OCD suffer from obsessions, defined by recurrent intrusive anxious thoughts or images, and/or compulsions, defined by repetitive behaviors or mental acts. Although the patients are usually aware of the senselessness of the compulsions they are performing, they are unable to inhibit their behaviors. Currently, two apparently opposing theories have been postulated to explain compulsivity in OCD. The classic cognitive-behavioral theory proposes that performing compulsions is primarily a strategy to reduce distress that is evoked by obsessions^2^. This theory provides an explanation of OCD symptoms that forms the basis of current cognitive-behavioral therapy. However, the variety in phenomenology shows that the relationship between obsessionality and compulsivity is more complex^3^. Recent neuroscientific studies suggest that patients with OCD show a general impairment in goal-directed behavior, resulting in an overreliance on habits^4,5^. Goal-directed behavior consists of actions that are performed to achieve a desired goal. When actions are performed on a regular basis, habits are formed to facilitate actions that do not require planning or organization. This leads to greater efficiency, but often at the cost of behavioral flexibility. Along these lines, the overreliance on (maladaptive) habitual behavior is thought to contribute to the development of compulsivity in OCD^6^.

Both rodent and human studies have pointed towards the involvement of the posterior putamen and the caudate nucleus in the habit and goal-directed network, respectively^6–10^. Both structures are part of the corticostriatal circuitry, the dysfunction of which is seen as the neuroanatomical basis of OCD^11,12^. While the posterior putamen mediates habitual actions by targeting motor areas including the supplementary motor area (SMA), goal directed behavior is mediated by the interaction between the ventral medial prefrontal cortex (vmPFC) and the caudate nucleus^9^. The recent focus on the habit hypothesis became apparent in the discussion around the new DSM-5, in which OCD moved out of the chapter ‘anxiety disorders’ to obtain its own chapter: i.e., ‘obsessive–compulsive and related disorders’^13^. The habit theory however, does not account for the typical clinical observation that psychological distress exacerbates OCD symptoms^14^. Furthermore, recent studies in healthy humans have shown that stress reduces prefrontal cortex-related goal-directed control and thereby induces a bias towards reliance on habitual behavior^15,16^. Integrating these findings leads to the hypothesis that psychological distress may reduce goal-directed control and induce a shift towards habits, thereby contributing to the compulsivity seen in OCD^6,17^. However, this novel model that connects the critical role of distress as posited by the classical cognitive-behavioral theory with the neuroscience-inspired habit theory of OCD remains to be tested.

In this study, we investigated the effects of psychological distress on resting-state functional connectivity of both the caudate nucleus (as major hub within the goal-directed network), and the posterior putamen (as major hub of the habit network) comparing OCD patients with matched healthy controls. The effects of stress vary over time due to the rapid release of neuromodulators and the delayed release of corticosteroids. In addition, corticosteroids also have time-dependent effects related to rapid non-genomic and slow non-genomic effects^18^. As a result, catecholaminergic and dopaminergic systems are activated directly after a stressor, while the slower genomic effects of corticosteroids can continue for several hours and affect cognitive and emotional processing^19–21^. Acute stress can thereby lead to psychological distress, which is defined as a negative state in which adaptation mechanisms fail to restore psychological homeostasis^22^. Because there was a delay between stress induction and scanning, we will further refer to the psychological changes after the stress induction as psychological distress. We hypothesized that in OCD patients, connectivity is reduced within the goal-directed network (i.e. between the caudate nucleus and the ventromedial prefrontal cortex) and/or increased within the habit network (i.e. between the posterior putamen and supplementary motor area) during psychological distress.

## Methods

### Participants

Twenty-five OCD patients were recruited from the outpatient clinic for anxiety disorders at the psychiatry department of the Amsterdam UMC and through advertisement at a Dutch patient organization website. Inclusion criteria were (1) being aged 18-65 years; (2) having a diagnosis of OCD according to the DSM-IV; (3) having a Yale-Brown Obsessive-Compulsive Scale (Y-BOCS) score of 12 or higher; (4) having provided written informed consent and being willing and able to understand, participate and comply with the study requirements. Exclusion criteria are reported in the Supporting Information. In addition, twenty-five healthy controls without a current or past psychiatric diagnosis as assessed with the M.I.N.I. and matched according to age, sex, and educational level, were recruited through flyers and online advertisements. All participants were assessed on anxiety, depressive and obsessive-compulsive symptoms, using the Hamilton Anxiety Rating Scale (HAM-A), the Hamilton Depression Rating Scale (HDRS)^23^ and the Y-BOCS^24^, respectively. In addition, the presence of psychiatric co-morbidities was assessed with the M.I.N.I.^25^ and the Structured Interview for DSM-IV Personality (SIDP-IV)^26^. The authors assert that all procedures contributing to this work comply with the ethical standards of the relevant national and institutional committees on human experimentation and with the Helsinki Declaration of 1975, as revised in 2008. All procedures involving human subjects/patients were approved by the Ethical Committee of the Academic Medical Center in Amsterdam (METC 2014_168) and all participants provided written informed consent. The study was registered in the ISRCTN registry (ISRCTN47698087).

### Design and measurements

**Fig. 1:**
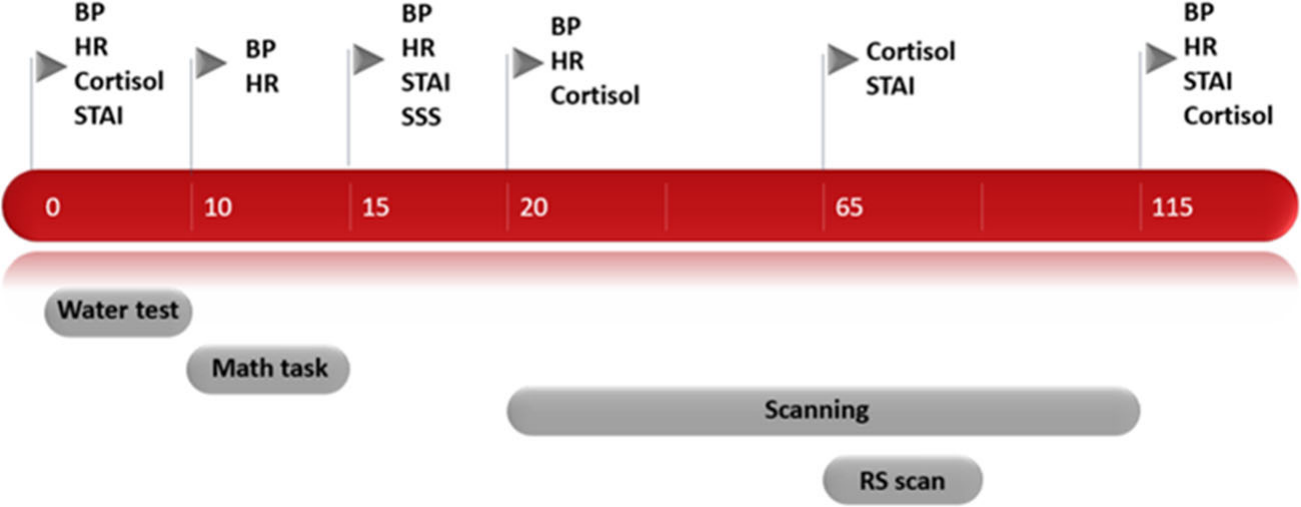
Timeline of experimental procedure and measurements. Abbreviations: blood pressure (BP), heart rate (HR), State-Trait Anxiety Inventory (STAI), subjective stress scale (SSS) and resting-state (RS) scan.

The study used a cross-over design with counterbalanced order of stress induction vs. neutral condition across participants, with the two sessions separated by a 1 week interval. All the sessions took place in the afternoon to control for the normal daily fluctuations of cortisol. During the stress session, participants were exposed to the socially evaluated cold-pressor test (SECPT). This is a relatively short, extensively studied procedure which is known to induce a reliable physiological and subjective stress response through a minimally invasive method^27^ (see Supplemental Information). The cold stress procedure causes vasoconstriction, which consequently leads to increased blood pressure and heart rate deceleration^27^. The physiological stress response was examined by measuring blood pressure and heart rate at five time points: at baseline, once during the water immersion procedure, after the math task, before the scanning session (45 min before the resting-state scan), and after the scanning session (40 min after the resting-state scan), see Fig. 1. In addition, cortisol was measured through saliva sampling at four time points: at baseline, after the math task, right before the resting-state scan, and after the scanning session. Moreover, to assess the subjective stress response, participants were asked to rate their anxiety level by filling out the State-Trait Anxiety Inventory (STAI^28^) at four time points: prior to the water procedure, after the math task, right before the resting-state scan, and after the scanning session. Furthermore, participants had to rate how difficult, unpleasant, and stressful they experienced the stress procedure right after the math task using a subjective stress level questionnaire with a VAS scale^27^. Because this study was part of a larger set of experiments, the resting-state scan was conducted approximately 65 min after the stress induction procedure. At the end of the stress session there was a short debriefing, in which the participants were told that the purpose of the test was to create stress. For an overview of the study design and measurements, see Fig. 1. Because previous studies have shown that twenty participants or more should be included in functional neuroimaging studies in order to have sufficient reliability we aimed to include at least twenty participants per group^29^.

### Data acquisition

MR imaging was performed on a 3.0 T Philips MRI scanner (Philips, Best, The Netherlands), using a 32-channel SENSE head coil. The scanning protocol (see Supplemental Information) included a high-resolution T1-weighted MRI (voxel size = 1.1 mm isotropic). For fMRI, multi-echo echoplanar imaging (EPI) was used to acquire T2*-weighted MRI volumes with blood oxygen level-dependent (BOLD) contrast (repetition time = 2375 ms; voxel size = 3.0 mm isotropic). Multi-echo EPI reduces signal dropout and distortion while enhancing BOLD contrast sensitivity^30^. Participants were instructed to stay awake and relax with their eyes open while thinking of anything that came to their mind.

### Data analysis

Demographical and clinical data were analyzed with SPSS software (version 24.0, Chicago, IL, USA). Group differences were tested using Mann-Whitney U tests for continues not normally distributed variables (age, Y-BOCS score, HAM-A, HDRS, mean FD) and *χ*^2^ tests for gender, handedness and education. The physiological stress measures (blood pressure, heart rate and cortisol) and anxiety levels (STAI) were analyzed using mixed model analysis of variance (ANOVA) with the factors group (patients, healthy controls), condition (stress, control) and time (five time points for blood pressure and heart rate, four for anxiety), corrected for the order of sessions. No time factor was used for cortisol, as we calculated the area under the curve with respect to the ground (AUCg) to assess overall exposure to cortisol during the session^31^. We additionally compared baseline cortisol values between the conditions to ensure that the results were not due to significant differences at baseline. Baseline cortisol and AUCg values were log-transformed before testing because of a non-normal distribution. Each question of the subjective stress level questionnaire was entered in a mixed model ANOVA with the factors factors group (patients, healthy controls), condition (stress, control) and question type (how difficult, how stressful, how unpleasant), corrected for the order of sessions. When the assumption of sphericity was violated the ANOVA results were corrected with a Greenhouse Geisser correction. The analyses were followed by post-hoc paired-t-testing in case of significance, with Bonferroni correction for multiple comparisons.

### MRI analysis

Standard preprocessing of functional MRI data was conducted in SPM12 (www.fil.ion.ucl.ac.uk/spm/) and the CONN toolbox v18 (http://www.nitrc.org/projects/conn) including realignment, slice-time correction, normalization to Montreal Neurological Institute (MNI) space and 6 mm smoothing (see Supplemental Information). Participants with realignment parameters exceeding 3 mm translation on the *x*-, *y*-, or *z*-axis were excluded from the analysis, resulting in the exclusion of two patients. One healthy control was excluded due to an incidental neurological finding and one control was excluded because of insufficient brain coverage due to inaccurate setting of the field of view. This led to the inclusion of twenty-three patients and twenty-three healthy controls for further analysis.

We assessed functional connectivity of the left and right caudate and left and right posterior putamen using a seed-to-voxel analysis to investigate whether there were group differences in distress-induced connectivity. Data were corrected for nuisance variables using CompCor^32^, band-pass filtered, and transformed using Fisher’s r-to-z transformation^33^. Because the caudate nucleus and posterior putamen are located closely to each other, the timeseries from the ipsilateral seed were regressed out to ensure that the results were specific for the selected seeds and did not reflect striatal connectivity in general. Connectivity maps were entered into a second-level group (patient, healthy control) X condition (stress, control) mixed model ANOVA, corrected for age, gender and the order of sessions (i.e. stress induction at first or second session). Post-hoc analyses were performed in case of significance to determine the direction of the interaction effect. In addition, we analyzed group differences during the control visit with a two sample t-test to assess whether patients showed altered connectivity without the presence of distress, again corrected for age, gender and the order of sessions. Voxel-wise statistical tests were family wise error (FWE) rate corrected (two-sided; *P* < 0.05) for multiple comparisons across the whole brain at cluster level using an initial height threshold of *P* < 0.001 with a small volume correction for the regions targeted by our seeds. Because we were most interested in connectivity within both hypothesized networks, we performed a small volume correction for the regions targeted by our seeds, i.e. the supplementary motor area (SMA) for the posterior putamen and the ventromedial prefrontal cortex (vmPFC) for the caudate nucleus. We used the WFU Pickatlas toolbox in SPM12 to define the SMA ROI according to the anatomical automatic labeling (AAL) atlas. For the goal-directed ROI, we selected coordinates of the vmPFC that have shown to be active during goal-directed behavior in an instrumental discrimination task using a sphere with 10 mm radius^34^. Movement during scanning was investigated by calculating the mean framewise displacement (FD) for each scanning session, and was compared between groups with a Mann-Whitney U test. To further investigate whether the imaging results were linked to the severity and type of symptoms, ratings on the obsession and compulsion scale of the YBOCS were correlated with the connectivity results extracted from the significant clusters using a Pearson correlation in SPSS (see Supplemental information).

## Results

### Demographic and clinical data

Demographic and clinical data are presented in Table 1. There were no significant group differences in age, sex distribution, level of education and movement during scanning. As expected, ratings on the Y-BOCS, HAM-A and HDRS were significantly higher in patients than in controls. Patients had various current comorbid disorders, including panic disorder (*n* = 2), agoraphobia (*n* = 1), hypochondria (*n* = 1), generalized anxiety disorder (*n* = 1), social anxiety disorder (*n* = 1), past major depressive disorder (*n* = 6) and past alcohol abuse (*n* = 1). In addition, three patients matched the criteria for a personality disorder, i.e. obsessive-compulsive, antisocial and avoidant personality disorder. Furthermore, nineteen patients used a stabile dose of medication during the two visits, including fluoxetine, (es)citalopram, sertraline, paroxetine, clomipramine or venlafaxine.

**Table 1:** Demographic and clinical data presented as the mean ± SD.

	Patients (*n* = 23)	Controls (*n* = 23)	*p*-Value
Age (years)	33.48 ± 2.02	33.52 ± 3.07	*p* = 0.605^1^
Gender (male/female)	10/13	12/11	*p* = 0.555^2^
Handedness (left/right)	2/21	4/19	*p* = 0.381^2^
Highest education (*n*)	3/5/7/3/5	0/4/10/4/5	*p* = 0.436^2^
Lower professional	3	0	
Secondary school	5	4	
Medium professional	7	10	
Higher professional	8	9	
Y-BOCS:			
Total	22.57 ± 1.24	0.13 ± 0.10	*p* < 0.001^1^*
Obsessions	11.61 ± 0.64	0.13 ± 0.10	
Compulsions	10.52 ± 0.84	0.00 ± 0.00	
HAM-A	11.83 ± 1.57	1.39 ± 1.64	*p* < 0.001^1^*
HDRS	8.17 ± 1.24	0.65 ± 0.19	*p* < 0.001^1^*
Medication status (yes/no)	19/4	0/23	
Framewise displacement (mm)			
Control visit	0.14 ± 0.02	0.12 ± 0.01	*p* = 0.538^1^
Stress visit	0.16 ± 0.03	0.12 ± 0.01	*p* = 0.070^1^

*Significant difference between groups, *p* ≤ 0.05, tested with ^1^Mann-Whitney *U*-test or ^2^chi-square test. Scales: Yale-Brown Obsessive-Compulsive Scale (Y-BOCS), Hamilton Anxiety Rating Scale (HAM-A), Hamilton Depression Rating Scale (HDRS).

### Stress response

**Fig. 2:**
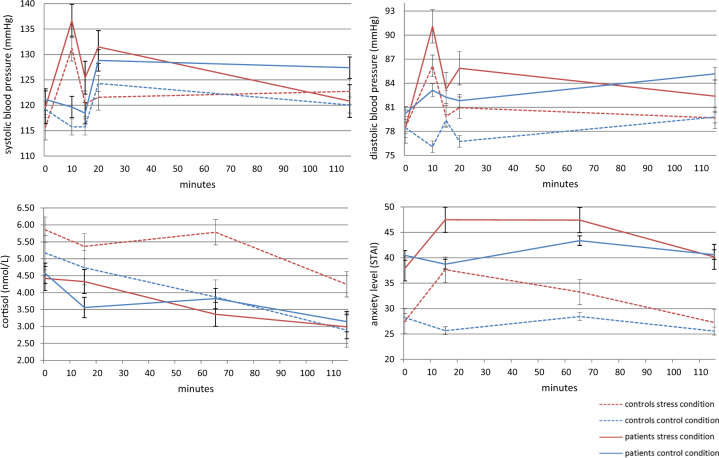
Physiological and subjective stress response, time points: baseline (0 min), after water test (10 min), after math task (15 min), before scanning session (20 min), before resting-state scan (65 min) and after scanning (115 min) reported as the mean with standard error.

Physiological and subjective stress responses are presented in Fig. 2. Blood pressure and heart rate were analyzed using a mixed model ANOVA with the factors group (patients, healthy controls), condition (stress, control) and time (five time points), corrected for the order of sessions. For systolic blood pressure, this analysis revealed a main effect of condition with higher systolic blood pressure in the stress condition (*F*(1,35) = 4.64, *p* = 0.038, *η*_p_^2^ = 0.12), a main effect of time (*F*(4,140) = 3.20, *p* = 0.015, *η*_p_^2^ = 0.08) and a condition X time interaction (*F*(4,140) = 8.91, *p* < 0.001, *η*_p_^2^ = 0.20). The condition × time interaction was due to higher systolic blood pressure after the math task during the stress condition compared to the control condition across groups (*t*(45) = 6.35, *p* < 0.001). The analysis of the diastolic blood pressure revealed a main effect of condition with higher diastolic blood pressure in the stress condition (*F*(1,34) = 4.24, *p* = 0.047, *η*_p_^2^ = 0.11). As expected, the analysis of the heart rate did not show any significant results^27^. For cortisol, the analysis of baseline level showed no significant effects. We then calculated the AUCg and entered these values into an interaction analysis with the factors group (patients, healthy controls) and condition (stress, control). The analysis revealed a group×condition interaction (*F*(1,43) = 6.36, *p* = 0.015, *η*_p_^2^ = 0.13). Post-hoc testing showed significantly higher cortisol levels in the stress condition than the control condition in healthy controls (*t*(22) = 3.83, *p* = 0.001) but not in patients. The analysis of subjective anxiety levels, as measured with the STAI, revealed a main effect of condition with higher anxiety levels in the stress condition (*F*(1,40) = 5.20, *p* = 0.028, *η*_p_^2^ = 0.12), a main effect of time (*F*(2.08,83.12) = 4.45, *p* = 0.014, *η*_p_^2^ = 0.10) and a condition X time interaction (*F*(2.61,104.28) = 12.31, *p* < 0.001, *η*_p_^2^ = 0.24). In addition, there was a significant main effect of group (*F*(1,40) = 46.3, *p* < 0.001, *η*_p_^2^ = 0.54), indicating that patients reported higher anxiety levels than healthy controls overall. Post-hoc paired T-testing showed that all participants reported significantly higher anxiety levels in the stress condition compared to the control condition after the stress procedure (*t*(45) = 7.17, *p* < 0.001), with a trend towards significance during scanning (*t*(43) = 2.22, *p* = 0.032). The analysis of the subjective stress level questionnaire showed a main effect of condition with higher ratings in the stress condition (*F*(1,42) = 105.99, *p* < 0.001, *η*_p_^2^ = 0.72). Analyzing the specific subscales showed that all participants rated the stress condition as more difficult (*t*(44) = 12.09, *p* < 0.001), more unpleasant (*t*(44) = 13.02, *p* < 0.001), and more stressful (*t*(44) = 10.8, *p* < 0.001) than the control condition. Together, these results show that the stress condition successfully increased blood pressure and psychological stress measures in both groups, and that group differences were only present for the stress-induced cortisol response, and anxiety levels (independent of stress induction).

### Seed-to-voxel analysis

**Fig. 3:**
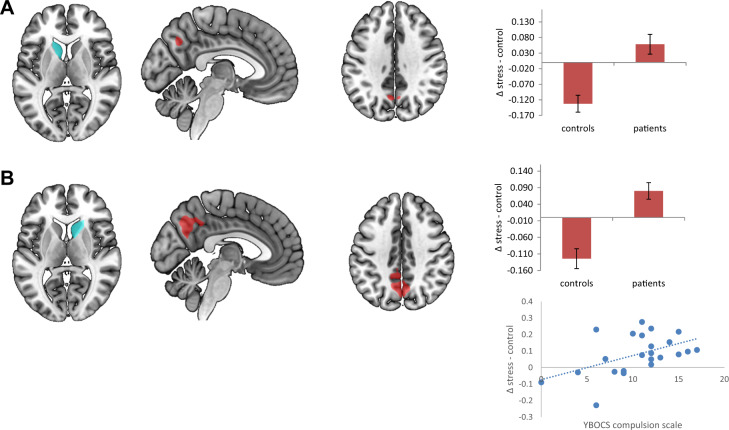
Stress effects in patients compared to healthy controls with the selected seeds, significant connectivity results (thresholded at *p* > 0.001 for illustrational purposes), contrast estimates extracted from the significant clusters, and correlation with compulsion scale of the YBOCS. **a** Seed region: left caudate nucleus; OCD patients >healthy controls (stress >control condition). **b** Seed region: right caudate nucleus; OCD patients >healthy controls (stress >control condition).

To assess the effects of distress on these networks, we performed a mixed model ANOVA with the factors group (patients, healthy controls) and condition (stress, control), corrected for the order of sessions. This analysis revealed a significant interaction for the goal-directed seeds (for significant clusters, see Table 2). Compared to healthy controls, patients showed a blunted decrease in connectivity between the left and right caudate nuclei and precuneus during psychological distress (see Fig. 3). This interaction effect was mainly driven by decreased connectivity in healthy controls between the right caudate nucleus and precuneus and the left caudate nucleus and precuneus. The increase in connectivity during distress in patients was not significant. We found no significant clusters in the vmPFC using a small volume correction. For the habit seeds, we found no significant group differences in connectivity during psychological distress between the posterior putamen and the SMA, nor with other regions of the brain. Furthermore, there was no main effect of distress across patients and controls in any of the seeds.

**Table 2:** Significant clusters from the seed-to-voxel group × condition interaction analysis.

Seed	MNI coordinates			Cluster size	*p*-Value	Region	BA
	*x*	*y*	*z*				
OCD patients > healthy controls (stress > control condition)							
Caudate nucleus L	−6	−62	+ 38	92	0.038	precuneus	7
Caudate nucleus R	−4	−66	+ 42	467	<0.001	precuneus	7
Posterior putamen L					no clusters		
Posterior putamen R					no clusters

We further investigated whether the connectivity results were linked to the severity and type of symptoms, by correlating the extracted values from the significant clusters of the stress versus control contrast with the ratings on the obsession and compulsion scale of the Y-BOCS in patients only. This showed a significant correlation between the connectivity of the right caudate nucleus and precuneus and ratings on the compulsion scale of the Y-BOCS (*r* = 0.49, *n* = 23, *p* = 0.017, see Fig. 3), indicating that patients who are more compulsive show a larger increase in connectivity during psychological distress. There were no significant correlations between the imaging results and the rating on the obsession scale of the Y-BOCS.

We then analyzed group differences during the control session with a two sample t-test to see whether patients showed altered connectivity without the presence of stress, corrected for age, gender and the order of sessions. Compared to healthy controls, patients showed significantly lower connectivity between the caudate nucleus and lateral occipital cortex, the angular gyrus, the middle and superior frontal gyrus, the precuneus cortex, the posterior cingulate gyrus and the frontal pole (*p*(FWE) < 0.05, for significant clusters see Supplemental Information Table 1. Both the left and right posterior putamen showed no significant differences in baseline connectivity between patients and controls.

To further understand the altered connectivity of the default mode network in patients, we performed a post-hoc analysis with the significant coordinates of the precuneus as seed region. Mixed model analysis with the factors group (patients, healthy controls) and condition (stress, control) showed a significant interaction between the precuneus and the intraparietal sulcus (IPS) (peak MNI: 26, -80, 42, cluster size: 264, *p*(FWE) < 0.001). Post-hoc one sample T-tests showed that the interaction during distress was driven by a non-significant decrease in connectivity in patients relative to a non-significant increase in connectivity in controls. Additionally, we analyzed group differences in the precuneus during the control session with a two sample *t*-test to see whether patients showed altered connectivity without the presence of stress, which showed no significant results.

## Discussion

This is the first study investigating the effects of distress on intrinsic functional connectivity in patients with OCD, which aimed to test the hypothesis that distress induces a shift from the goal-directed towards the habit network. Using a seed-to-voxel resting-state analysis, we found a blunted reduction in connectivity between the seeds involved in the goal-directed network (i.e., the left and right caudate nucleus) and the precuneus in patients with OCD during psychological distress. This result was positively correlated with the severity of compulsive symptoms. Distress did not have an effect on functional connectivity between the seeds involved in the habit network (i.e., the left and right posterior putamen) and the rest of the brain, including the supplementary motor areas. Measures of blood pressure and subjective anxiety confirmed successful stress induction across groups, and showed significantly lower cortisol responses in patients compared to healthy controls. Together, these results suggest that distress-induced compulsivity is primarily associated with recruitment of the goal-directed network, rather than with changes in the habit network. The habit theory of OCD is centered around the balance between the goal-directed and habit networks, but ignores the influence of distress. Distress however, is a central aspect of the cognitive-behavioral theory of OCD. Although we found no evidence that distress leads to an overdependence on the habit network, the result of altered goal-directed connectivity does link the apparent contradictory theories of OCD and suggest that both theories contribute to the pathophysiology of OCD.

Our results were not fully in line with our hypothesis, as distress did not affect intrinsic connectivity within the habit network, and we did not observe reduced connectivity of the goal-directed network with the ventromedial prefrontal cortex. Instead, the results point towards altered connectivity between the caudate nucleus and the precuneus, an area that is part of the default mode network (DMN). The precuneus is involved in self-consciousness and self-referential processing, defined as the process of relating information to the self^35,36^. When individuals are not involved in an attention demanding or goal-directed task, the DMN is activated and self-referential processing is believed to predominate^37^. Compared to healthy controls, patients showed decreased baseline connectivity between the goal-directed network and the precuneus. During distress, healthy controls showed uncoupling of the goal-directed network and DMN, which implies that distress normally induces upscaling of the goal-directed network at the cost of the DMN. In contrast, OCD patients did not show a decrease in connectivity between the caudate nucleus and precuneus during distress, suggesting that patients might be stuck in self-referential processing at the cost of goal-directed behavior. Almost all OCD symptoms can be narrowed down to the underlying desire for absolute certainty and maintaining control, therefore obsessions usually develop in domains where there is no absolute level of certainty, such as sexuality, sickness and danger^3^. Unfortunately patients do not base the level of certainty on external reality, but on feelings of anxiousness and unrealistic thoughts. This causes them to continuously focus on themselves, resulting in a mental loop of self-referential processing. The lack of downscaling of the DMN that was seen in patients might thus be the result of compulsive self-referential processing during psychological distress. Post-hoc analysis showed a significant interaction between the precuneus and the IPS, with relatively decreased coupling of the precuneus and IPS during distress in patients. The IPS is part of the dorsal attention network and is associated with voluntary attention, which is defined as selectively allocating the processing resources to goal-relevant locations in the visual field. The dorsal attention network is usually activated during goal-directed task performance and is therefore suggested to be anticorrelated with the default mode network under normal conditions^38^. The findings of decreased coupling of the precuneus and IPS under distress in patients could therefore imply that voluntary goal-directed attention to external stimuli is also hampered by DMN dominance. This could further support the hypothesis that patients are predominantly internally focused during distress at the cost of goal-directed behavior. The distress-induced connectivity between the right caudate nucleus and the precuneus was positively correlated with the compulsion scale of the Y-BOCS, indicating that patients who are prone to compulsive behavior are more likely to show a blunted reduction in connectivity during distress. This further confirms the assumption that our results can be interpreted as self-referential processing during distress at the cost of goal-directed control, resulting in mental compulsions. Our findings further substantiates the current treatment of choice, i.e., cognitive behavioral therapy, in which performing compulsions is seen as a strategy to reduce distress that is evoked by obsessions. Furthermore, current therapies might benefit from focusing more on attention training techniques to learn how to shift the focus away from their thoughts. This way, the self-referential processing can be attenuated resulting in enhancement of executive control over cognitive processing.

Remarkably, we found no distress induced coupling between the posterior putamen and SMA. This leads to the speculation that compulsions are more strongly related to impaired goal-directed control than to overdependence on habits. Our null finding could be due to the study design, as the motor circuit is not probed during resting-state. Future research is needed to see whether these results are confirmed using a task that requires goal-directed and habitual behavior for further understanding of the pathophysiology of OCD. Furthermore, we cannot rule out that there were early effects of stress that are missed because of the delay between the stress induction and the resting-state scan. This study was part of a larger trial, therefore the resting-state scan was conducted ~65 min after the SECPT. Although patients were no longer in the acute stress state, cortisol levels in the control group were still elevated (Fig. 2), and research has shown that the effects of corticosteroids can continue for several hours^19^. Additionally, participants still rated their anxiety level to be higher before the resting-state scan started in the stress visit compared to the control visit, implicating that the participants were experiencing psychological distress during the scanning session. In addition to the group differences in connectivity, patients also showed lower stress-induced cortisol levels compared to healthy controls. This blunted cortisol response could be due to adaptation of stress circuits as a consequence of chronic exposure to stress. This mechanism is seen across multiple psychiatric disorders including post-traumatic stress disorder, major depressive disorder and panic disorder^39^. Similarly, previous studies have also shown a non-response of cortisol during stressful exposure and response prevention therapy in OCD^40,41^.

Without the presence of distress, the goal-directed seeds showed extensive group differences in connectivity with frontal areas, whereas the habit seeds showed no differences in connectivity. This is consistent with the hypothesis that patients already show an impairment in the goal-directed network, regardless of the presence of induced distress^4^. The finding of reduced connectivity between the caudate and these cortical regions is inconsistent with corticostriatal hyperconnectivity that is often described in OCD^42–44^, but has previously been demonstrated in research conducted in unmedicated patients^45^.

This study has several limitations. Resting-state fMRI is a promising technique for investigating the functional architecture of the brain, but the results provide no information on the direction of the connectivity. Therefore, we can only speculate on causal relationships between significant nodes^46^. As mentioned above, we might have missed stress-induced effects within the habit network in the acute stress phase due to the delay between the stress induction and the resting-state scan. Last, the majority of our patients were using medication and had various comorbid disorders which could have influenced the imaging results. Despite these limitations, our results show that psychological distress influences functional connectivity of the goal-directed network in OCD. These findings provide first insight into the effects of distress on the balance between the goal-directed and habit network in OCD, implicating that distress-induced compulsivity is predominantly associated with involvement of the goal-directed network.

## Supplementary information

Supplemental information

Supplemental table
