# Supplementary material for: The effect of distress on the balance between goal-directed and habit networks in obsessive-compulsive disorder
Source: Transl Psychiatry. 2020 Feb 24;10:73. doi: 10.1038/s41398-020-0744-7 (PMC7039895; doi:10.1038/s41398-020-0744-7)
Supplement: Supplementary file 2 — Supplemental table [file 41398_2020_744_MOESM2_ESM.docx]

TABLE 1; Significant clusters in baseline connectivity comparison between patients and controls.

| Seed | MNI coordinates | | | Cluster size | P-value | Region | BA |
| --- | --- | --- | --- | --- | --- | --- | --- |
|  | x | y | z |  | (FWE) |  |  |
| Healthy controls > OCD patients (control condition only) | | | | | | | |
| Caudate nucleus L | -46 | -60 | +36 | 477 | <0.001 | lateral occipital cortex L, angular gyrus L | 39 |
|  | -34 | +24 | +50 | 357 | <0.001 | mid. and sup. frontal gyrus L | 8 |
|  | -6 | -64 | +38 | 267 | <0.001 | precuneus cortex | 7 |
|  | -4 | -40 | +26 | 243 | <0.001 | posterior cingulate gyrus | 26 |
|  | +14 | +64 | +22 | 183 | 0.002 | frontal pole R | 10 |
|  | +28 | +18 | +40 | 106 | 0.037 | mid. and sup. frontal gyrus R | 9 |
| Caudate nucleus R | -30 | +16 | +58 | 321 | <0.001 | mid. and sup. frontal gyrus L | 8 |
|  | -2 | -66 | +30 | 204 | <0.001 | precuneus cortex | 7 |
|  | -4 | -48 | +26 | 196 | 0.001 | posterior cingulate gyrus | 30 |
|  | -38 | -58 | +30 | 190 | 0.002 | lateral occipital cortex L, angular gyrus L | 39 |
|  | -12 | +58 | +20 | 161 | 0.004 | frontal pole L | 10 |
| Posterior putamen L |  |  |  |  | N.S. |  |  |
| Posterior putamen R |  |  |  |  | N.S. |  |  |
